# 1-[(*Z*)-(5-Methyl-2-pyrid­yl)iminiometh­yl]-2-naphtholate

**DOI:** 10.1107/S1600536810000346

**Published:** 2010-01-09

**Authors:** Xin-Yu Liu, Yu-Hua Fan, Qiang Wang, Cai-Feng Bi, Yu-Fang Wang

**Affiliations:** a Key Laboratory of Marine Chemistry Theory and Technology, Ministry of Education, College of Chemistry and Chemical Engineering, Ocean University of China, Qingdao, Shandong 266100, People’s Republic of China

## Abstract

In the zwitterionic title compound, C_17_H_14_N_2_O, the dihedral angle between the naphthalene and pyridine ring systems is 3.56 (9)° and an intra­molecular N—H⋯O hydrogen bond generates an *S*(6) ring. In the crystal, mol­ecules are linked by C—H⋯O inter­actions.

## Related literature

For a related structure, see: Eltayeb *et al.* (2007 ▶).
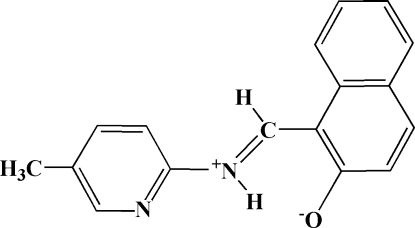


## Experimental

### 

#### Crystal data

C_17_H_14_N_2_O
*M* *_r_* = 262.30
Monoclinic, 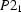

*a* = 4.8703 (2) Å
*b* = 9.5525 (5) Å
*c* = 14.0804 (6) Å
β = 98.353 (2)°
*V* = 648.12 (5) Å^3^
*Z* = 2
Mo *K*α radiation
μ = 0.09 mm^−1^
*T* = 296 K
0.47 × 0.10 × 0.09 mm

#### Data collection

Bruker APEXII CCD diffractometer
Absorption correction: multi-scan (*SADABS*; Bruker, 2005 ▶) *T*_min_ = 0.961, *T*_max_ = 0.992
6930 measured reflections
1660 independent reflections
1321 reflections with *I* > 2σ(*I*)
*R*_int_ = 0.029

#### Refinement

*R*[*F* ^2^ > 2σ(*F* ^2^)] = 0.038
*wR*(*F* ^2^) = 0.109
*S* = 0.98
1660 reflections
182 parameters
1 restraint
H-atom parameters constrained
Δρ_max_ = 0.18 e Å^−3^
Δρ_min_ = −0.15 e Å^−3^

### 

Data collection: *APEX2* (Bruker, 2005 ▶); cell refinement: *SAINT* (Bruker, 2005 ▶); data reduction: *SAINT*; program(s) used to solve structure: *SHELXS97* (Sheldrick, 2008 ▶); program(s) used to refine structure: *SHELXL97* (Sheldrick, 2008 ▶); molecular graphics: *SHELXTL* (Sheldrick, 2008 ▶); software used to prepare material for publication: *SHELXL97*.

## Supplementary Material

Crystal structure: contains datablocks I, global. DOI: 10.1107/S1600536810000346/hb5298sup1.cif

Structure factors: contains datablocks I. DOI: 10.1107/S1600536810000346/hb5298Isup2.hkl

Additional supplementary materials:  crystallographic information; 3D view; checkCIF report

## Figures and Tables

**Table 1:** Hydrogen-bond geometry (Å, °)

*D*—H⋯*A*	*D*—H	H⋯*A*	*D*⋯*A*	*D*—H⋯*A*
N2—H2⋯O1	0.86	1.89	2.571 (3)	135
C3—H3⋯O1^i^	0.93	2.46	3.346 (3)	159

Symmetry code: (i) 
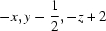
.

**Fig. 1.**
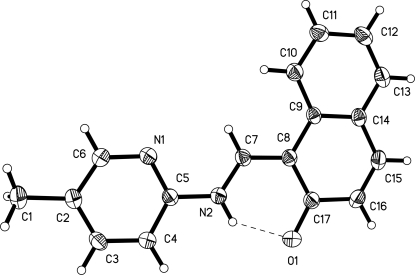
The structure of (I) showing 30% probability displacement ellipsoids.

**Fig. 2.**
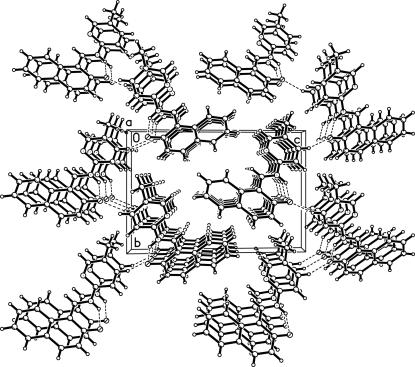
The crystal packing of (I), viewed approximately along the a axis.

## supplementary crystallographic information

### Comment

One similar compound as 1-{2-[(2-hydroxy-1-naphthyl)methyleneamino] phenyliminiomethyl}-2-naphtholate methanol hemisolvate has been synthesized and characterized by X-ray diffraction (Eltayeb *et al.*, 2007). We now report on the title compound, (I), whose structure was determined by X-ray diffraction (Fig. 1).

This compound, which has a non-planar molecular structure, contains two aromatic rings linked through a imine group. The dihedral angle between the two aromatic rings C2—C3—C4—C5—N1—C6 and C8—C9—C14—C15—C16—C17 is 3.46(0.16)°. Intramolecular N—H···O hydrogen bonds are observed in the molecular structure, similar to those reported structure (Eltayeb *et al.*, 2007). The molecules is formed a one-dimensional zigzag chain through intermolecular C—H···O hydrogen bonds, which make the molecule more stabile.

As seen in Fig. 2, the molecules are linked into a one-dimensional chain by intermolecular C—H···O hydrogen bonds (Table 2).

### Experimental

2-Hydroxy-1-naphthaldehyde (1 mmol, 172.2 mg) were added with stirring to anhydrous ethanol (30 ml) to make a solution. It was slowly dropped into the anhydrous ethanol solution (15 ml) containing (1 mmol, 108.1 mg) 5-methylpyridin-2-amine at 339 K and mixture was stirred at 339 K for 4 h, a mass of deep yellow grain was separated out. The product was collected by filtration and washed several times with anhydrous ethanol and dried under vacuum. Yellow needles of (I) were obtained by slow evaporation at room temperature from anhydrous ethanol solution after 4 days.

### Refinement

Anomalous dispersion was negligible and Friedel pairs were merged before refinement. All H-atoms were positioned geometrically (C—H = 0.93–0.96Å, N—H = 0.86Å) and refined as riding with *U*_iso_(H) =1.2*U*_eq_(carrier).

### Crystal data

**Table:**

C_17_H_14_N_2_O	*F*(000) = 276
*M**_r_* = 262.30	*D*_x_ = 1.344 Mg m^−^^3^
Monoclinic, *P*2_1_	Mo *K*α radiation, λ = 0.71073 Å
Hall symbol: P 2yb	Cell parameters from 1877 reflections
*a* = 4.8703 (2) Å	θ = 2.6–25.6°
*b* = 9.5525 (5) Å	µ = 0.09 mm^−^^1^
*c* = 14.0804 (6) Å	*T* = 296 K
β = 98.353 (2)°	Needle, yellow
*V* = 648.12 (5) Å^3^	0.47 × 0.10 × 0.09 mm
*Z* = 2	

### Data collection

**Table:**

Bruker APEXII CCD diffractometer	1660 independent reflections
Radiation source: fine-focus sealed tube	1321 reflections with *I* > 2σ(*I*)
graphite	*R*_int_ = 0.029
φ and ω scans	θ_max_ = 28.1°, θ_min_ = 2.6°
Absorption correction: multi-scan (*SADABS*; Bruker, 2005)	*h* = −6→6
*T*_min_ = 0.961, *T*_max_ = 0.992	*k* = −12→11
6930 measured reflections	*l* = −18→15

### Refinement

**Table:**

Refinement on *F*^2^	Primary atom site location: structure-invariant direct methods
Least-squares matrix: full	Secondary atom site location: difference Fourier map
*R*[*F*^2^ > 2σ(*F*^2^)] = 0.038	Hydrogen site location: inferred from neighbouring sites
*wR*(*F*^2^) = 0.109	H-atom parameters constrained
*S* = 0.98	*w* = 1/[σ^2^(*F*_o_^2^) + (0.062*P*)^2^ + 0.0757*P*] where *P* = (*F*_o_^2^ + 2*F*_c_^2^)/3
1660 reflections	(Δ/σ)_max_ < 0.001
182 parameters	Δρ_max_ = 0.18 e Å^−^^3^
1 restraint	Δρ_min_ = −0.15 e Å^−^^3^

### Special details

**Table:**

Geometry. All e.s.d.'s (except the e.s.d. in the dihedral angle between two l.s. planes) are estimated using the full covariance matrix. The cell e.s.d.'s are taken into account individually in the estimation of e.s.d.'s in distances, angles and torsion angles; correlations between e.s.d.'s in cell parameters are only used when they are defined by crystal symmetry. An approximate (isotropic) treatment of cell e.s.d.'s is used for estimating e.s.d.'s involving l.s. planes.
Refinement. Refinement of *F*^2^ against ALL reflections. The weighted *R*-factor *wR* and goodness of fit *S* are based on *F*^2^, conventional *R*-factors *R* are based on *F*, with *F* set to zero for negative *F*^2^. The threshold expression of *F*^2^ > σ(*F*^2^) is used only for calculating *R*-factors(gt) *etc*. and is not relevant to the choice of reflections for refinement. *R*-factors based on *F*^2^ are statistically about twice as large as those based on *F*, and *R*- factors based on ALL data will be even larger.

### Fractional atomic coordinates and isotropic or equivalent isotropic displacement parameters (Å^2^)

**Table:**

	*x*	*y*	*z*	*U*_iso_*/*U*_eq_	
C1	−0.5458 (5)	−0.0508 (3)	0.8827 (2)	0.0561 (6)	
H1A	−0.6558	−0.0735	0.8224	0.084*	
H1B	−0.6646	−0.0178	0.9266	0.084*	
H1C	−0.4484	−0.1328	0.9086	0.084*	
C2	−0.3401 (5)	0.0616 (2)	0.86752 (16)	0.0431 (5)	
C3	−0.2411 (5)	0.1558 (3)	0.93834 (17)	0.0518 (6)	
H3	−0.3011	0.1508	0.9980	0.062*	
C4	−0.0533 (5)	0.2576 (3)	0.92116 (16)	0.0492 (6)	
H4	0.0127	0.3223	0.9683	0.059*	
C5	0.0341 (4)	0.2610 (2)	0.83229 (15)	0.0382 (5)	
C6	−0.2414 (5)	0.0762 (3)	0.78108 (16)	0.0478 (6)	
H6	−0.3082	0.0143	0.7322	0.057*	
C7	0.3255 (4)	0.3752 (2)	0.73144 (15)	0.0398 (5)	
H7	0.2584	0.3151	0.6815	0.048*	
C8	0.5254 (4)	0.4749 (2)	0.71574 (15)	0.0383 (5)	
C9	0.6227 (4)	0.4846 (2)	0.62325 (15)	0.0371 (5)	
C10	0.5226 (5)	0.3996 (3)	0.54463 (16)	0.0468 (6)	
H10	0.3905	0.3315	0.5518	0.056*	
C11	0.6141 (5)	0.4140 (3)	0.45734 (16)	0.0501 (6)	
H11	0.5423	0.3566	0.4064	0.060*	
C12	0.8133 (5)	0.5138 (3)	0.44477 (17)	0.0525 (6)	
H12	0.8743	0.5238	0.3855	0.063*	
C13	0.9182 (5)	0.5968 (3)	0.51987 (18)	0.0510 (6)	
H13	1.0539	0.6623	0.5116	0.061*	
C14	0.8257 (4)	0.5857 (2)	0.60997 (16)	0.0413 (5)	
C15	0.9323 (5)	0.6740 (3)	0.68904 (18)	0.0499 (6)	
H15	1.0677	0.7395	0.6804	0.060*	
C16	0.8457 (5)	0.6663 (3)	0.77458 (18)	0.0484 (6)	
H16	0.9224	0.7259	0.8236	0.058*	
C17	0.6352 (5)	0.5673 (2)	0.79247 (16)	0.0430 (5)	
N1	−0.0571 (4)	0.1724 (2)	0.76194 (13)	0.0462 (5)	
N2	0.2284 (4)	0.3625 (2)	0.81358 (13)	0.0424 (4)	
H2	0.2877	0.4203	0.8588	0.051*	
O1	0.5562 (4)	0.5643 (2)	0.87507 (11)	0.0567 (5)	

### Atomic displacement parameters (Å^2^)

**Table:**

	*U*^11^	*U*^22^	*U*^33^	*U*^12^	*U*^13^	*U*^23^
C1	0.0517 (14)	0.0467 (15)	0.0725 (16)	−0.0048 (12)	0.0176 (12)	0.0080 (13)
C2	0.0391 (10)	0.0397 (12)	0.0520 (12)	0.0037 (10)	0.0115 (9)	0.0067 (11)
C3	0.0604 (14)	0.0536 (15)	0.0459 (12)	−0.0031 (13)	0.0230 (11)	0.0044 (12)
C4	0.0581 (14)	0.0484 (14)	0.0438 (12)	−0.0078 (12)	0.0163 (10)	−0.0060 (11)
C5	0.0403 (10)	0.0341 (11)	0.0416 (11)	0.0036 (9)	0.0108 (8)	0.0026 (9)
C6	0.0525 (13)	0.0435 (13)	0.0478 (12)	−0.0049 (11)	0.0081 (10)	−0.0027 (11)
C7	0.0436 (11)	0.0356 (11)	0.0418 (11)	0.0025 (9)	0.0116 (8)	0.0036 (9)
C8	0.0411 (10)	0.0322 (11)	0.0429 (11)	0.0033 (9)	0.0106 (8)	0.0024 (9)
C9	0.0370 (10)	0.0337 (11)	0.0421 (10)	0.0038 (9)	0.0103 (8)	0.0058 (9)
C10	0.0496 (13)	0.0447 (14)	0.0481 (12)	−0.0016 (11)	0.0136 (10)	0.0000 (11)
C11	0.0555 (14)	0.0529 (15)	0.0437 (12)	0.0054 (12)	0.0135 (10)	−0.0005 (11)
C12	0.0590 (14)	0.0553 (15)	0.0475 (13)	0.0092 (12)	0.0225 (10)	0.0091 (11)
C13	0.0504 (13)	0.0447 (14)	0.0615 (15)	0.0012 (11)	0.0203 (11)	0.0108 (12)
C14	0.0404 (11)	0.0356 (12)	0.0493 (12)	0.0033 (10)	0.0113 (9)	0.0055 (10)
C15	0.0486 (12)	0.0385 (13)	0.0645 (14)	−0.0057 (11)	0.0145 (10)	0.0025 (12)
C16	0.0508 (13)	0.0365 (12)	0.0582 (14)	−0.0035 (11)	0.0086 (10)	−0.0055 (11)
C17	0.0492 (12)	0.0356 (12)	0.0460 (12)	0.0033 (10)	0.0125 (9)	0.0016 (10)
N1	0.0536 (11)	0.0441 (11)	0.0430 (10)	−0.0029 (10)	0.0146 (8)	−0.0014 (9)
N2	0.0497 (11)	0.0370 (10)	0.0425 (9)	−0.0017 (9)	0.0134 (8)	−0.0010 (8)
O1	0.0721 (11)	0.0548 (11)	0.0463 (9)	−0.0073 (10)	0.0187 (8)	−0.0083 (8)

### Geometric parameters (Å, °)

**Table:**

C1—C2	1.504 (3)	C8—C9	1.452 (3)
C1—H1A	0.9600	C9—C10	1.402 (3)
C1—H1B	0.9600	C9—C14	1.413 (3)
C1—H1C	0.9600	C10—C11	1.374 (3)
C2—C3	1.377 (4)	C10—H10	0.9300
C2—C6	1.379 (3)	C11—C12	1.390 (4)
C3—C4	1.380 (4)	C11—H11	0.9300
C3—H3	0.9300	C12—C13	1.360 (4)
C4—C5	1.379 (3)	C12—H12	0.9300
C4—H4	0.9300	C13—C14	1.410 (3)
C5—N1	1.329 (3)	C13—H13	0.9300
C5—N2	1.407 (3)	C14—C15	1.433 (3)
C6—N1	1.339 (3)	C15—C16	1.335 (3)
C6—H6	0.9300	C15—H15	0.9300
C7—N2	1.317 (3)	C16—C17	1.444 (3)
C7—C8	1.402 (3)	C16—H16	0.9300
C7—H7	0.9300	C17—O1	1.277 (3)
C8—C17	1.436 (3)	N2—H2	0.8600
			
C2—C1—H1A	109.5	C14—C9—C8	119.20 (19)
C2—C1—H1B	109.5	C11—C10—C9	121.8 (2)
H1A—C1—H1B	109.5	C11—C10—H10	119.1
C2—C1—H1C	109.5	C9—C10—H10	119.1
H1A—C1—H1C	109.5	C10—C11—C12	120.4 (2)
H1B—C1—H1C	109.5	C10—C11—H11	119.8
C3—C2—C6	116.3 (2)	C12—C11—H11	119.8
C3—C2—C1	122.3 (2)	C13—C12—C11	119.4 (2)
C6—C2—C1	121.5 (2)	C13—C12—H12	120.3
C2—C3—C4	120.3 (2)	C11—C12—H12	120.3
C2—C3—H3	119.8	C12—C13—C14	121.5 (2)
C4—C3—H3	119.8	C12—C13—H13	119.3
C5—C4—C3	118.4 (2)	C14—C13—H13	119.3
C5—C4—H4	120.8	C13—C14—C9	119.4 (2)
C3—C4—H4	120.8	C13—C14—C15	121.8 (2)
N1—C5—C4	123.2 (2)	C9—C14—C15	118.72 (19)
N1—C5—N2	117.42 (18)	C16—C15—C14	122.9 (2)
C4—C5—N2	119.4 (2)	C16—C15—H15	118.6
N1—C6—C2	125.2 (2)	C14—C15—H15	118.6
N1—C6—H6	117.4	C15—C16—C17	121.3 (2)
C2—C6—H6	117.4	C15—C16—H16	119.4
N2—C7—C8	123.2 (2)	C17—C16—H16	119.4
N2—C7—H7	118.4	O1—C17—C8	122.9 (2)
C8—C7—H7	118.4	O1—C17—C16	119.3 (2)
C7—C8—C17	119.32 (18)	C8—C17—C16	117.86 (19)
C7—C8—C9	120.61 (19)	C5—N1—C6	116.61 (19)
C17—C8—C9	120.07 (19)	C7—N2—C5	124.41 (19)
C10—C9—C14	117.45 (19)	C7—N2—H2	117.8
C10—C9—C8	123.34 (19)	C5—N2—H2	117.8
			
C6—C2—C3—C4	−0.1 (4)	C10—C9—C14—C13	0.0 (3)
C1—C2—C3—C4	−179.7 (2)	C8—C9—C14—C13	−179.1 (2)
C2—C3—C4—C5	−0.8 (4)	C10—C9—C14—C15	−179.9 (2)
C3—C4—C5—N1	1.1 (4)	C8—C9—C14—C15	1.0 (3)
C3—C4—C5—N2	−179.1 (2)	C13—C14—C15—C16	179.2 (2)
C3—C2—C6—N1	0.9 (4)	C9—C14—C15—C16	−0.9 (4)
C1—C2—C6—N1	−179.5 (2)	C14—C15—C16—C17	−0.3 (4)
N2—C7—C8—C17	1.0 (3)	C7—C8—C17—O1	−1.0 (3)
N2—C7—C8—C9	−179.45 (19)	C9—C8—C17—O1	179.5 (2)
C7—C8—C9—C10	1.4 (3)	C7—C8—C17—C16	178.5 (2)
C17—C8—C9—C10	−179.1 (2)	C9—C8—C17—C16	−1.1 (3)
C7—C8—C9—C14	−179.55 (19)	C15—C16—C17—O1	−179.3 (2)
C17—C8—C9—C14	0.0 (3)	C15—C16—C17—C8	1.2 (3)
C14—C9—C10—C11	−0.8 (3)	C4—C5—N1—C6	−0.3 (3)
C8—C9—C10—C11	178.3 (2)	N2—C5—N1—C6	179.8 (2)
C9—C10—C11—C12	0.6 (4)	C2—C6—N1—C5	−0.7 (3)
C10—C11—C12—C13	0.4 (4)	C8—C7—N2—C5	−178.1 (2)
C11—C12—C13—C14	−1.2 (4)	N1—C5—N2—C7	−0.1 (3)
C12—C13—C14—C9	1.0 (3)	C4—C5—N2—C7	−180.0 (2)
C12—C13—C14—C15	−179.1 (2)		

### Hydrogen-bond geometry (Å, °)

**Table:**

*D*—H···*A*	*D*—H	H···*A*	*D*···*A*	*D*—H···*A*
N2—H2···O1	0.86	1.89	2.571 (3)	135
C3—H3···O1^i^	0.93	2.46	3.346 (3)	159

Symmetry codes: (i) −*x*, *y*−1/2, −*z*+2.
